# A 3′UTR modification of the TNF-α mouse gene increases peripheral TNF-α and modulates the Alzheimer-like phenotype in 5XFAD mice

**DOI:** 10.1038/s41598-020-65378-2

**Published:** 2020-05-26

**Authors:** Nikoleta Kalovyrna, Olympia Apokotou, Sotiria Boulekou, Evi Paouri, Athena Boutou, Spiros Georgopoulos

**Affiliations:** Laboratory of Cellular Neurobiology, Center of Basic Research, Biomedical Research Foundation, Academy of Athens, 11527 Athens, Greece

**Keywords:** Neuroscience, Neuroimmunology

## Abstract

Tumor necrosis factor-α (TNF-α) is a pro-inflammatory cytokine, involved in Alzheimer’s disease pathogenesis. Anti-TNF-α therapeutic approaches currently used in autoimmune diseases have been proposed as a therapeutic strategy in AD. We have previously examined the role of TNF-α and anti-TNF-α drugs in AD, using 5XFAD mice, and we have found a significant role for peripheral TNF-α in brain inflammation. Here we investigated the role of mouse TNF-α on the AD-like phenotype of 5XFAD mice using a knock-in mouse with deletion of the 3’UTR of the endogenous TNF-α (TNF^ΔARE/+^) that develops rheumatoid arthritis and Crohn’s disease. 5XFAD/TNF^ΔARE/+^ mice showed significantly decreased amyloid deposition. Interestingly, microglia but not astrocytes were activated in 5XFAD/ TNF^ΔARE/+^ brains. This microglial activation was associated with increased infiltrating peripheral leukocytes and perivascular macrophages and synaptic degeneration. APP levels and APP processing enzymes involved in Aβ production remained unchanged, suggesting that the reduced amyloid burden can be attributed to the increased microglial and perivascular macrophage activation caused by TNF-α. Peripheral TNF-α levels were increased while brain TNF-α remained the same. These data provide further evidence for peripheral TNF-α as a mediator of inflammation between the periphery and the brain.

## Introduction

Alzheimer’s disease (AD) is the most common cause of dementia among the elderly characterized by severe memory loss and cognitive impairment^1^. Although beta-amyloid (Αβ) is traditionally accepted as the main initiator of the disease, numerous failed clinical trials targeting Αβ^2^ have changed research focus on other factors involved in the disease as brain inflammation, commonly called neuroinflammation^3,4^. Moreover, clinical and experimental studies have provided significant evidence that peripheral inflammation may be associated with increased neuroinflammation and accelerated AD progression. A number of pro-inflammatory molecules including TNF-α, have been implicated in AD pathogenetic process^5^. TNF-α has been shown to colocalize with amyloid plaques in AD human brains and animal models^6,7^ and elevated TNF-α levels have been detected in the serum and cerebrospinal fluid (CSF) of AD patients^6,8,9^. TNF-α is a pleiotropic pro-inflammatory cytokine that mediates its effect through two different receptors, TNF-α receptor I (p55) and TNF-α receptor II (p75)^10^. TNF-α central role in the pathogenesis of chronic autoimmune diseases like rheumatoid arthritis (RA), psoriasis and Crohn’s disease^11–14^ has led to the development of anti-TNF-α therapeutics widely used in the treatment of autoimmune diseases^15,16^. Case studies have reported that treatment of AD patients with the anti-TNF-α agents currently used in RA or Crohn’s disease, was beneficial and improved cognitive impairment^17–19^ while epidemiological studies showed that AD risk is reduced in RA patients receiving anti-TNF-α agents^20^.

We have previously shown that peripheral human TNF-α and anti-TNF-α therapy, currently used in RA treatment, play a significant role in modulating neuroinflammation, amyloid deposition and neuronal degeneration in AD mice^21,22^. 5XFAD mice, carrying a 3’-UTR modified human TNF-α (hu TNF-α) transgene^23^ (5XFAD/Tg197) that results in increased peripheral expression of huTNF-α protein, develop an AD-like phenotype along with TNF-induced inflammatory arthritis, which can be suppressed by peripheral treatment with infliximab, a monoclonal anti-huTNF-α antibody widely used for RA treatment in patients^24^. 5XFAD/Tg197 mice exhibit robust brain inflammation, with extensive microglial and astrocytic activation and increased meningeal and perivascular macrophage activation that compromises neuronal integrity and synaptic health, despite reduced amyloid deposition. Interestingly, these alterations in the 5XFAD/Tg197 brains, correlated with increased levels of peripheral human TNF-α, suggesting that peripheral TNF-α alone is able to modulate brain inflammation through a TNF-α dependent periphery-to-brain communication pathway.

To further elucidate the role of the TNF signaling pathway and evaluate the effect of the modulation of endogenous mouse TNF-α gene (muTNF-α), in the current study, we employed the TNF^ΔARE/+^ mouse model and generated 5XFAD/TNF^ΔARE/+^ mice. In TNF^ΔARE/+^ mice deletion of the 3’-UTR of the endogenous mouse gene (muTNF-α) results in increased peripheral expression of the muTNF-α and the development of autoimmune inflammatory disease^25^. Analysis of 5XFAD/TNF^ΔARE/+^ mice revealed increased peripheral muTNF-α levels, reduced amyloid deposition, activated microglia and increased perivascular macrophages and infiltrating leukocytes. 5XFAD/TNF^ΔARE/+^ brains showed increased synaptic degeneration. Interestingly astrocytes were not activated, while APP processing enzymes remained unchanged. Our results provide further evidence for an association between systemic peripheral inflammation and AD pathogenesis and suggest peripheral TNF-α as a central link.

## Results

### 5ΧFAD/TNF^ΔARE/+^ mice have elevated TNF-α protein levels in the periphery only and not in the brain

**Figure 1:**
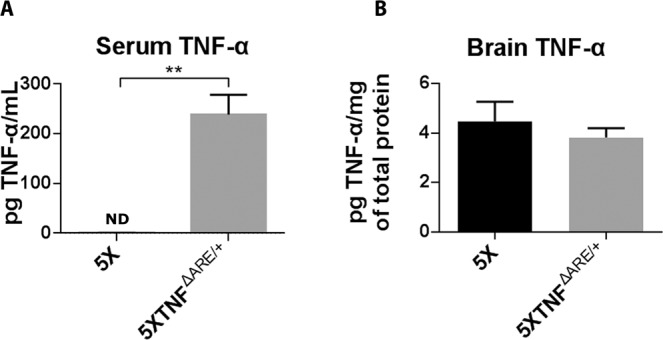
Peripheral muTNF-α is increased in 5XFAD/TNF^ΔARE/+^ mice, whereas brain muTNF-α remains unchanged. Quantitation of muTNF-α levels using ELISA in the serum (**A**) and brain (**B**) of 4-month-old 5XFAD/TNF^ΔARE/+^ and 5XFAD control mice. 5XFAD/TNF^ΔARE/+^ mice show significantly increased serum TNF-α (**A**) compared to 5XFAD, whereas brain TNF-α levels shows no difference between the two groups (**B**). Data are mean ± SEM; *n* = 3–5 per group. **p = 0.040 (two-tailed unpaired t test) (ND = Not detected).

Modification of the endogenous muTNF-α gene in the TNF^ΔARE/+^ mice has been shown to significantly increase muTNF-α protein levels in the periphery^25^. To examine the effect of the modification of the endogenous muTNF-α gene and upregulation of muTNF-α levels in the 5XFAD/TNF^ΔARE/+^ mice, we measured muTNF-α protein levels in the serum and the brain of the mice using an anti-muTNF-α specific ELISA. Our analysis detected significantly elevated muTNF-α protein levels in the sera of 5XFAD/TNF^ΔARE/+^ mice (>200 pg/mL) while muTNF-α was not detected in 5XFAD sera (Fig. 1A). muTNF-α was also detected in low levels (~4 pg/mg total protein) in brain protein extracts from 5XFAD/TNF^ΔARE/+^ and 5XFAD control mice but showed no difference between the two groups (Fig. 1B). These results suggest that the modification of the endogenous muTNF-α gene in the 5XFAD/TNF^ΔARE/+^ mice has a major effect on the TNF-α levels of the periphery compared to the brain where expression of TNF-α is significantly lower compared to the periphery and shows no difference between 5XFAD/TNF^ΔARE/+^ and 5XFAD mice.

### Amyloid deposits and plaque load are reduced in the 5XFAD/TNF^ΔARE/+^ mouse brains

To evaluate the effects on the amyloid phenotype of AD mice, from the genetic modification of the endogenous muTNF-α gene, we used the 5XFAD transgenic mice^26^ which bear five familial AD mutations, three for the APP and two for the presenilin 1 gene, and develop an amyloid phenotype with amyloid deposition at 2 months of age and robust glial activation. 5XFAD mice were mated with, muTNF-α mutated mice lacking the AU-rich elements of TNF (TNF^ΔARE^)^25^ to generate 5XFAD/TNF^ΔARE/+^ mice. In this study we used heterozygous TNF^ΔARE/+^ mice, as homozygous (TNF^ΔARE/ΔARE^) develop a wasting phenotype and die early^27^. 5XFAD littermates were used as controls. We used only female mice for analysis, as male 5XFAD transgenic mice develop the amyloid phenotype with a two-month delay compared to females^26,28^.

**Figure 2:**
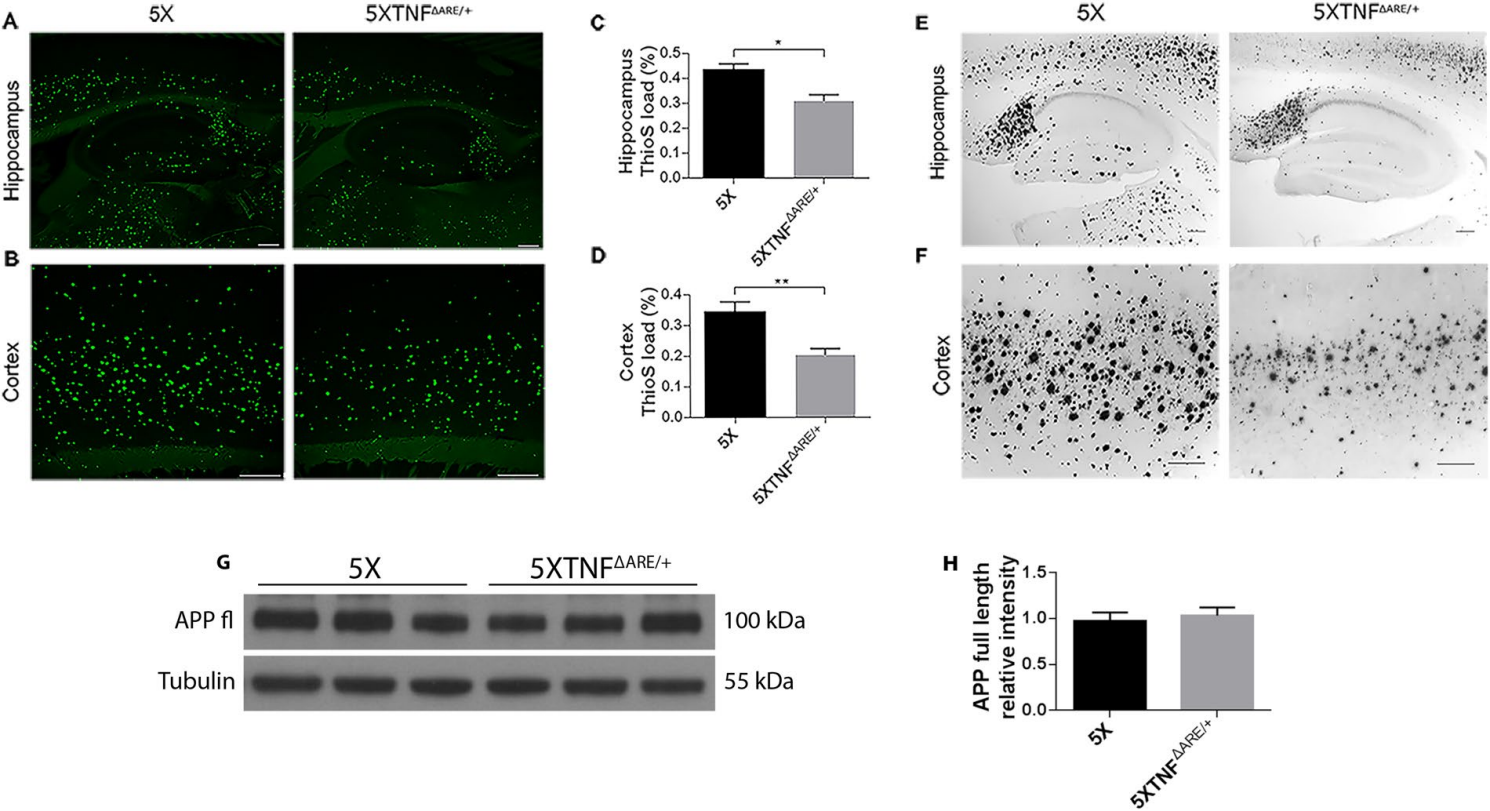
Genetic modification of the muTNF-α gene significantly reduces amyloid plaque load and Aβ deposition in 5XFAD/TNF^ΔARE/+^ mice. (**A,B**) Thioflavine-S staining of 40μm sagittal brain sections of 4-month-old 5XFAD and 5XFAD/TNF^ΔARE/+^ mice was performed to detect fibrillary amyloid plaques. Amyloid plaques are reduced in the hippocampus and cortex of 5XFAD/TNF^ΔARE/+^ mice compared to the 5XFAD. Representative pictures of the hippocampus **(A)** and the cortex **(B)** are shown for each mouse group. Scale bars: 250μm. (**C,D**) Quantitation of Thioflavine-S positive amyloid plaque load in the hippocampi **(C)** and cortices **(D)** of 4-month-old 5XFAD and 5XFAD/TNF^ΔARE/+^ mice is expressed as the percent area of positive staining. Analysis was performed using ImageJ software. Data are shown as mean ± SEM; *n* = 3–5 mice per group. For statistical analyses, two-tailed unpaired *t* test was used. *p = 0.0162; **p = 0.0077. **(E, F)** Immunohistochemistry for total Aβ (6E10 antibody) in 40μm sagittal brain sections of 4-month-old 5XFAD and 5XFAD/TNF^ΔARE/+^ mice. 5XFAD/TNF^ΔARE/+^ mice display significantly reduced Aβ deposition compared to the 5XFAD both in the hippocampus (**E**) and the cortex (**F**). Representative pictures of the hippocampus and cortex are shown for each mouse group. Scale bars: 250μm. (**G**) Western blot analysis of full-length APP brain protein levels in 4-month-old 5XFAD and 5XFAD/TNF^ΔARE/+^ mice showed no difference among the analyzed groups. (**H**) Quantitation was performed with densitometric analysis using ImageJ software. Data are mean ± SEM; *n* = 3 mice per group; experiment repeated 3 times using different samples. For statistical analyses, two-tailed unpaired *t* test was used.

40μm sagittal brain sections were stained with Thioflavine-S, to evaluate differences in amyloid plaque load between 5XFAD/TNF^ΔARE/+^ and 5XFAD 4-month-old mice. Confocal microscope image analysis revealed a significant reduction in Thioflavine-S positive amyloid plaque burden in the hippocampus and the cortex of 4-month-old 5XFAD/TNF^ΔARE/+^ mice compared to the 5XFAD (Fig. 2A,B). Thioflavine-S positive staining quantitation in the hippocampus confirmed a statistically significant decrease in 5XFAD/TNF^ΔARE/+^ mice compared to the 5XFAD (Fig. 2C). Similarly, quantitation of Thioflavine-S staining in the cortex also confirmed a statistically significant decrease (Fig. 2D).

To further examine the observed differences in fibrillary amyloid deposits, we performed immunohistochemistry in 40μm sagittal brain sections of 4-month-old 5XFAD and 5XFAD/TNF^ΔARE/+^ mice using the 6E10 antibody that detects both fibrillary and non-fibrillary Aβ. Αβ immunoreactivity in 5XFAD/TNF^ΔARE/+^ mice had a similar pattern to the Thioflavine-S positive plaques with sparsely distributed deposits in the cortex and accumulation mostly in the subiculum of the hippocampus (Fig. 2E,F). Together these results demonstrate that the genetic modification of TNF-α endogenous gene in the 5XFAD/TNF^ΔARE/+^ mouse brains results in reduced amyloid plaque burden and Αβ deposition. The observed plaque reduction in 5XFAD/TNF^ΔARE/+^ mice could potentially result from reduced APP levels and decreased Aβ production and amyloid plaque formation. To test this hypothesis, we evaluated the levels of full-length APP in total brain protein extracts from 5XFAD and 5XFAD/TNF^ΔARE/+^ mice by Western blotting (Fig. 2G). Subsequent analysis showed no significant difference of full-length APP protein levels among the 2 mouse groups (Fig. 2H), suggesting that the reduction of amyloid burden in 5XFAD/TNF^ΔARE/+^ mice is not caused by decreased production of APP.

### Modification of the muTNF-α endogenous gene does not alter APP processing enzymes in 5XFAD/TNF^ΔARE/+^ mice

**Figure 3:**
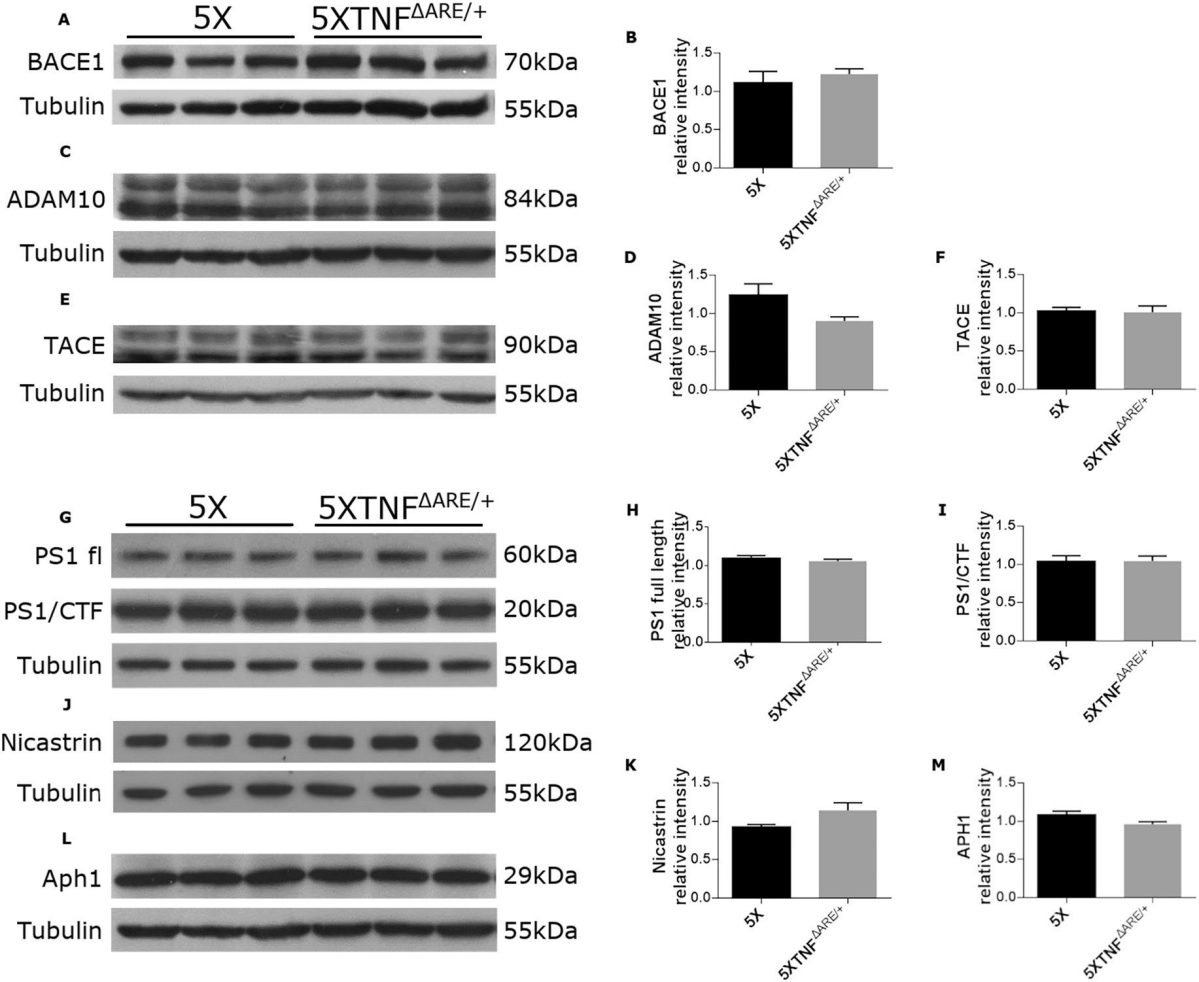
Genetic modification of the muTNF-α gene has no effect on APP processing enzymes. **(A-F)** Western blot analysis of BACE1, ADAM10 and TACE brain protein levels in 4-month-old 5XFAD/TNF^ΔARE/+^ and 5XFAD mice show no significant changes in levels of BACE1 (**A,B**), ADAM10 (**C,D**) and TACE (**E,F**), between the two groups. (**G**) Western blot analysis of full-length (fl) PS1 and PS1-CTFs protein levels in 4-month-old 5XFAD/TNF^ΔARE/+^ and 5XFAD mice. (**H,I**) Both PS1-fl and PS1-CTF protein levels show no difference between the two groups. (**J,L**) Western blot analysis of Nicastrin and Aph-1 protein levels in 4-month-old 5XFAD/TNF^ΔARE/+^ and 5XFAD mouse brains shows no significant difference between the two groups **(K, M).** Quantitation was performed with densitometric analysis using ImageJ software. Data represent mean ± SEM; *n* = 3 mice per group; experiment repeated 3 times. For statistical analyses, two-tailed unpaired *t* test was used.

A possible mechanism that accounts for the reduced amyloid deposition in the 5XFAD/TNF^ΔARE/+^ brains is by modulating APP processing and Aβ production. To examine whether the reduced amyloid plaques in 5XFAD/TNF^ΔARE/+^ mouse brains are caused by changes in APP metabolism, we quantified the brain protein levels of the APP processing enzymes that are involved in Aβ production. To evaluate the effect of muTNF-α 3′UTR modification on the APP processing enzymes we examined and quantified protein levels of key enzymes involved in APP processing and Aβ production, as TACE, BACE1 and ADAM10, as well as enzymes that constitute the γ-secretase complex as presenilin 1 (PS1), Nicastrin and Aph1. 5ΧFAD/TNF^ΔARE/+^ and 5XFAD total brain protein extracts were analyzed by Western blot analysis using specific antibodies to detect the above APP processing proteins. Since TNF-α is the main substrate of TACE, we examined whether muTNF-α gene modification can affect TACE protein levels in 5XFAD/TNF^ΔARE/+^ mice. Our analysis confirmed that TACE protein levels remained the same between the two groups (Fig. 3E,F). Similarly, ADAM10 and BACE1, the α- and β-secretases involved in APP processing and Aβ production did not show any significant change in their protein levels between the two groups (Fig. 3A-D).

TNF-α has also been involved in the regulation of γ-secretase cleavage of APP^29,30^. To examine whether TNF-α deficiency in 5XFAD mice affects the γ-secretase complex, we measured the levels of its catalytic subunit PS1, as well as Nicastrin and Aph1. Western blot analysis of total brain protein extracts from 5XFAD/TNF^ΔARE/+^ and 5ΧFAD control mice showed no difference in the levels of full-length (fl) PS1 between the two groups (Fig. 3G,H). Similarly, no differences were observed in PS1-CTFs between the two groups (Fig. 3G,I). Quantitation of Nicastrin and Aph1 protein levels also showed no difference between 5XFAD/TNF^ΔARE/+^ and 5ΧFAD control mice (Fig. 3J-M).

Overall these data provide evidence that muTNF-α gene modification in the 5XFAD/TNF^ΔARE/+^ mice does not affect the enzymes that mediate APP cleavage and Aβ production and suggests that TNF-α can be involved in modulating amyloid deposition in a different way.

### 5ΧFAD/TNF^ΔARE/+^ and TNF^ΔARE/+^ mice have activated microglia but not activated astrocytes

**Figure 4:**
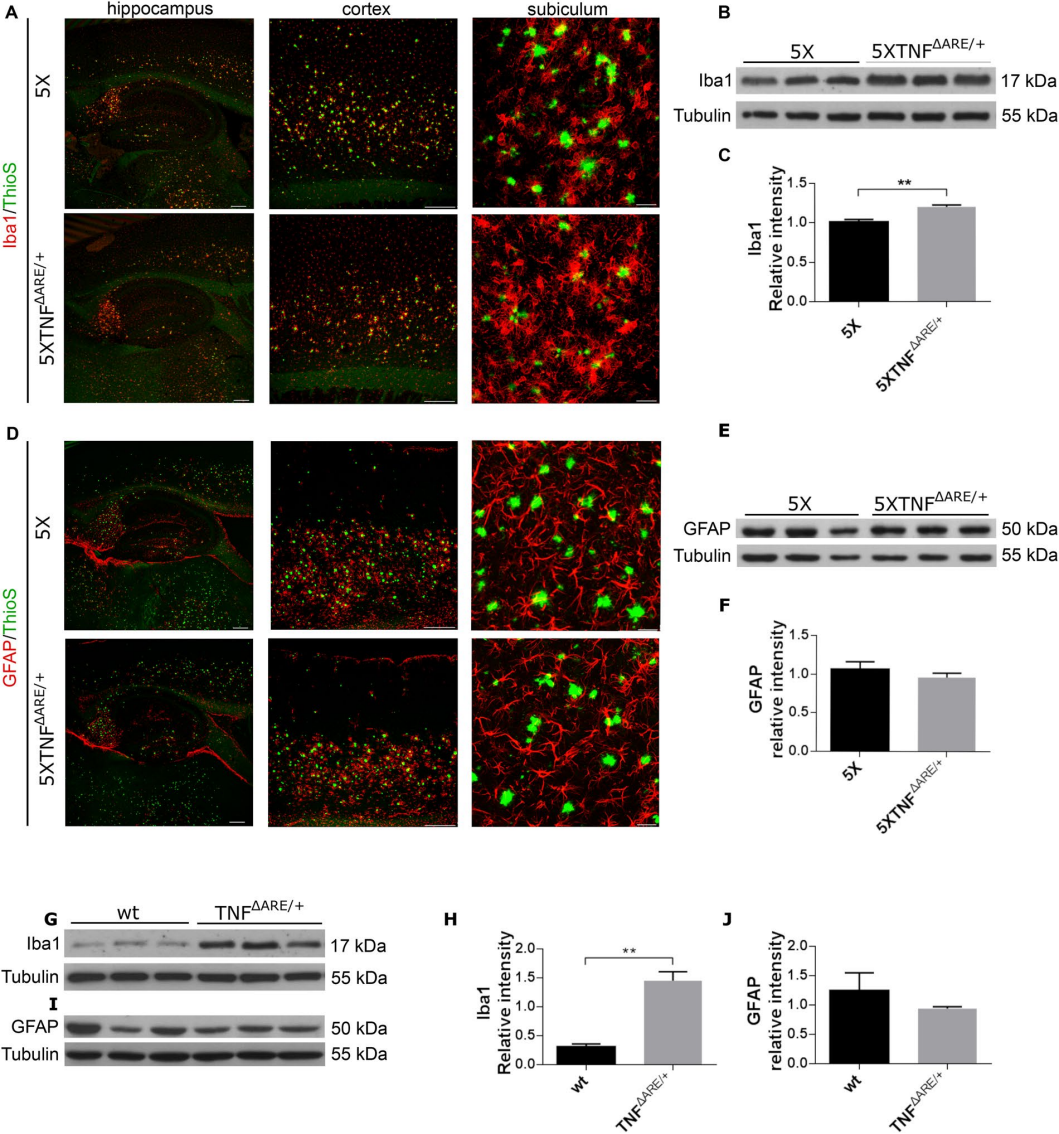
Microglia but not astrocytes are activated in 5XFAD/TNF^ΔARE/+^ mice. (**A,D**) Immunofluorescent detection of Iba1-positive microglia (**A**) and GFAP-positive astrocytes (**D**) in 40μm sagittal brain sections of 4-month-old 5XFAD/TNF^ΔARE/+^ and 5XFAD control mice. Sections were also stained with Thioflavine-S for amyloid plaques. 5XFAD/TNF^ΔARE/+^ brains display increased microglial cells surrounding amyloid plaques compared to 5XFAD brains. Representative pictures of the hippocampus, the cortex and the subiculum of the hippocampus are shown for each group. Scale bars: 250μm (hippocampus and cortex); 25μm (subiculum). (**B,C,E,F**) Western blot analysis shows significantly increased Iba1 brain protein levels (**B,C**) but not GFAP (**E,F**) in 5XFAD/TNF^ΔARE/+^ mice compared to 5XFAD mice. (**G,H,I,J**) Similarly, Western blot analysis of TNF^ΔARE/+^ and control brains, show significantly increased Iba1 (**G,H**) but not GFAP (**I,J**) brain protein levels in the TNF^ΔARE/+^ brain protein extracts compared to the C57BL/6 control brains. Quantitation was performed with densitometric analysis using ImageJ software. Data represent mean ± SEM; *n* = 3 mice per group; experiments repeated 3 times. For statistical analyses, two-tailed unpaired *t* test was used. **p = 0.0078 (C), **p = 0,0016 **(H)**.

To evaluate the effect of the 3′UTR modification of the muTNF-α endogenous gene and the increased levels of TNF-α in the periphery on the activation of glial cells, we analyzed 5ΧFAD/TNF^ΔARE/+^ and 5XFAD control brains for the microglial Iba1 marker and the astrocytic GFAP marker. Confocal analysis of 40μm sagittal brain sections double-stained for Iba1 and Thioflavine S showed a significant increase of reactive microglia in 5ΧFAD/TNF^ΔARE/+^ brains (Fig. 4A) compared to age-matched 5XFAD brains both in the cortex and the hippocampus of the brain. We observed an increase in the number of activated microglial cells surrounding amyloid plaques, as well as increased infiltration into the amyloid deposits, in the subiculum of the hippocampus of 5ΧFAD/TNF^ΔARE/+^ brains compared to 5XFAD (Fig. 4A). To further confirm this finding and quantify the increase of Iba1 protein, we performed Western blot analysis on total brain protein extracts from 5ΧFAD/TNF^ΔARE/+^ and 5XFAD mice. Analysis of Iba1 protein levels confirmed a statistically significant increase of Iba1 levels in 5ΧFAD/TNF^ΔARE/+^ brains compared to 5XFAD controls, in agreement with the increased microglial activation observed with immunofluorescence (Fig. 4B,C). To evaluate whether this increase in microglial activation, observed in the 5ΧFAD/TNF^ΔARE/+^ brains, is related to TNF^ΔARE/+^ phenotype, we analyzed Iba1 protein levels in the brain of TNF^ΔARE/+^ mice in comparison to wild-type control mice (Fig. 4G,H). Our analysis showed a statistically significant increase in Iba1 levels in the brains of TNF^ΔARE/+^ mice (Fig. 4H) suggesting an increase in microglial activation in TNF^ΔARE/+^ brains.

Next, we examined astrocytic activation in 5ΧFAD/TNF^ΔARE/+^ and 5XFAD mouse brains. Confocal analysis of 40μm sagittal brain sections double-stained for GFAP and Thioflavine-S showed no significant differences in reactive astrocytes surrounding amyloid plaques in 5ΧFAD/TNF^ΔARE/+^ mice compared to 5XFAD mice (Fig. 4D). To further confirm this finding and quantify GFAP protein levels, we performed Western blot analysis on total brain protein extracts from 5ΧFAD/TNF^ΔARE/+^ and 5XFAD mice. Similarly, to the immunofluorescence GFAP analysis, we did not observe any differences in GFAP protein levels between the two groups (Fig. 4E,F). Further analysis of GFAP protein levels with Western blot on TNF^ΔARE/+^ brains and wild-type controls also revealed no differences (Fig. 4I,J).

In summary, these data demonstrate that modification of the muTNF-α gene in the 5ΧFAD/TNF^ΔARE/+^ and TNF^ΔARE/+^ mice, and the increase of peripheral muTNF-α protein levels induce a robust microglial activation. This activation seems to be microglia specific as we did not observe any difference in astrocytic activation using the GFAP marker, both in the TNF^ΔARE/+^ and 5ΧFAD/TNF^ΔARE/+^ brains.

### 5XFAD/TNF^ΔARE/+^ mice display increased CD45 infiltrating leukocytes and CD68-positive phagocytic microglia as well as elevated LC3II levels in the brain

**Figure 5:**
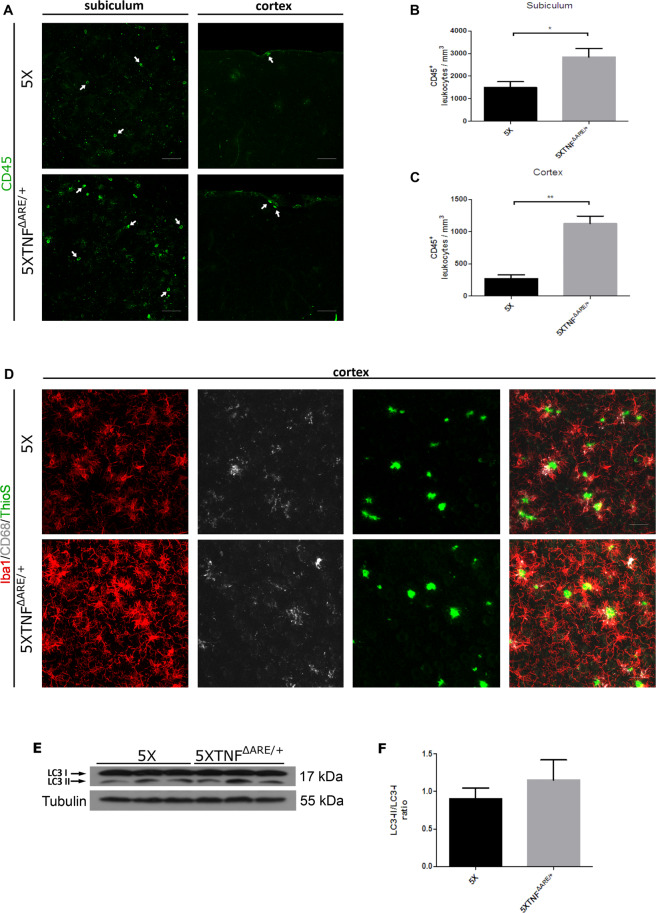
CD45 infiltrating leukocytes are increased while CD68-positive phagocytic microglia and LC3II levels show a trend towards increase in the 5XFAD/TNF^ΔARE/+^ brains. (**A,B,C**) Immunofluorescent detection of CD45 positive leukocytes at the subiculum of the hippocampus and the cortex (arrows) shows an increase in 5XFAD/TNF^ΔARE/+^ brains compared to 5XFAD controls. Representative pictures of the subiculum and the cortex (**A**) are shown for each group. Scale bars: 50 μm. Quantitation of CD45 leukocytes in the subiculum (**B**) and the cortex (**C**), reveals a significant increase in 5XFAD/TNF^ΔARE/+^ brains. Data are mean ± SEM; *n* = 3 mice per group. For statistical analyses, two-tailed unpaired *t* test was used. *p = 0,0269, **p = 0,0030 (**C**). (**D**) Immunofluorescent detection of CD68/Iba1 in brain sections shows a small non-significant increase of CD68 in activated microglia surrounding amyloid plaques in 5XFAD/TNF^ΔARE/+^ mice compared to 5XFAD controls. Sections were also stained for amyloid plaques with Thioflavine S. Scale bars: 50 μm. (**E**) Western blot analysis of LC3 brain protein levels in 5XFAD/TNF^ΔARE/+^ brains reveals an increase in the LC3II form and the LC3II/LC3I ratio (**F**), suggesting an increase in autophagy compared to 5XFAD controls. Analysis was performed with densitometric analysis using ImageJ software. Data are mean ± SEM; *n* = 3 mice per group. For statistical analyses, two-tailed unpaired *t* test was used.

Amyloid deposition, peripheral inflammation and/or arthritis have been implicated in the recruitment and infiltration of CD45-positive cells in the mouse and human AD brain^21,31–34^. To evaluate a similar effect in 5XFAD/TNF^ΔARE/+^ mice, brain sections were analyzed with confocal immunofluorescence microscopy for the presence of CD45-positive cells at the subiculum of the hippocampus and the meninges of the cortex. Our analysis revealed increased numbers of CD45-positive round-shaped leukocytes in the subiculum of the hippocampus of 5XFAD/TNF^ΔARE/+^ brains as well as in the cortex compared to 5XFAD control mice (Fig. 5A-C, arrows). The recruitment of peripheral leukocytes coincides with activated microglia and increased peripheral TNF-α levels and suggest for a potential cellular mechanism of inflammatory signal communication from the periphery to the brain.

The increased microglial activation and the reduced amyloid deposition observed in 5XFAD/TNF^ΔARE/+^ brains suggest for increased microglial-mediated Αβ clearance mechanisms. To examine this hypothesis, we immunostained for CD68, a lysosomal marker^35^ expressed in microglia associated with Aβ phagocytosis^36,37^, in 5XFAD/TNF^ΔARE/+^ and control brains. Confocal analysis revealed a slight non significant increase of CD68 immunoreactivity in microglia surrounding amyloid plaques in 5XFAD/TNF^ΔARE/+^ brains compared to 5XFAD controls (Fig. 5D). Next we evaluated LC3 levels in 5XFAD/TNF^ΔARE/+^ and control brains. LC3-associated phagocytosis has been connected to immune regulation and inflammation^38^. LC3 has also been implicated in the clearance mechanisms involved in Aβ removal and neurodegeneration in 5XFAD mice^39^. To examine whether alterations in LC3 related autophagy are involved in the decreased amyloid deposition in 5XFAD/TNF^ΔARE/+^ brains, we evaluated protein levels of LC3 and the ratio between the LC3II and the LC3I forms of the protein which are indicative of increased autophagy.Western blot analysis in total brain protein extracts from 5XFAD/TNF^ΔARE/+^ and 5XFAD control mice revealed a non-statistically significant increase in the levels of the LC3II form, providing evidence for a trend towards increase, in autophagy in the 5XFAD/TNF^ΔARE/+^ brains (Fig. 5E,F).

Overall, these results suggest that peripheral TNF-α in the 5XFAD/TNF^ΔARE/+^ mice controls the recruitment of CD45 positive leukocytes in the brain and in addition is able to regulate the phagocytic capacity of microglia against Aβ.

### 5ΧFAD/TNF^ΔARE/+^ mice show increased CD206-positive leptomeningeal and perivascular macrophages

**Figure 6:**
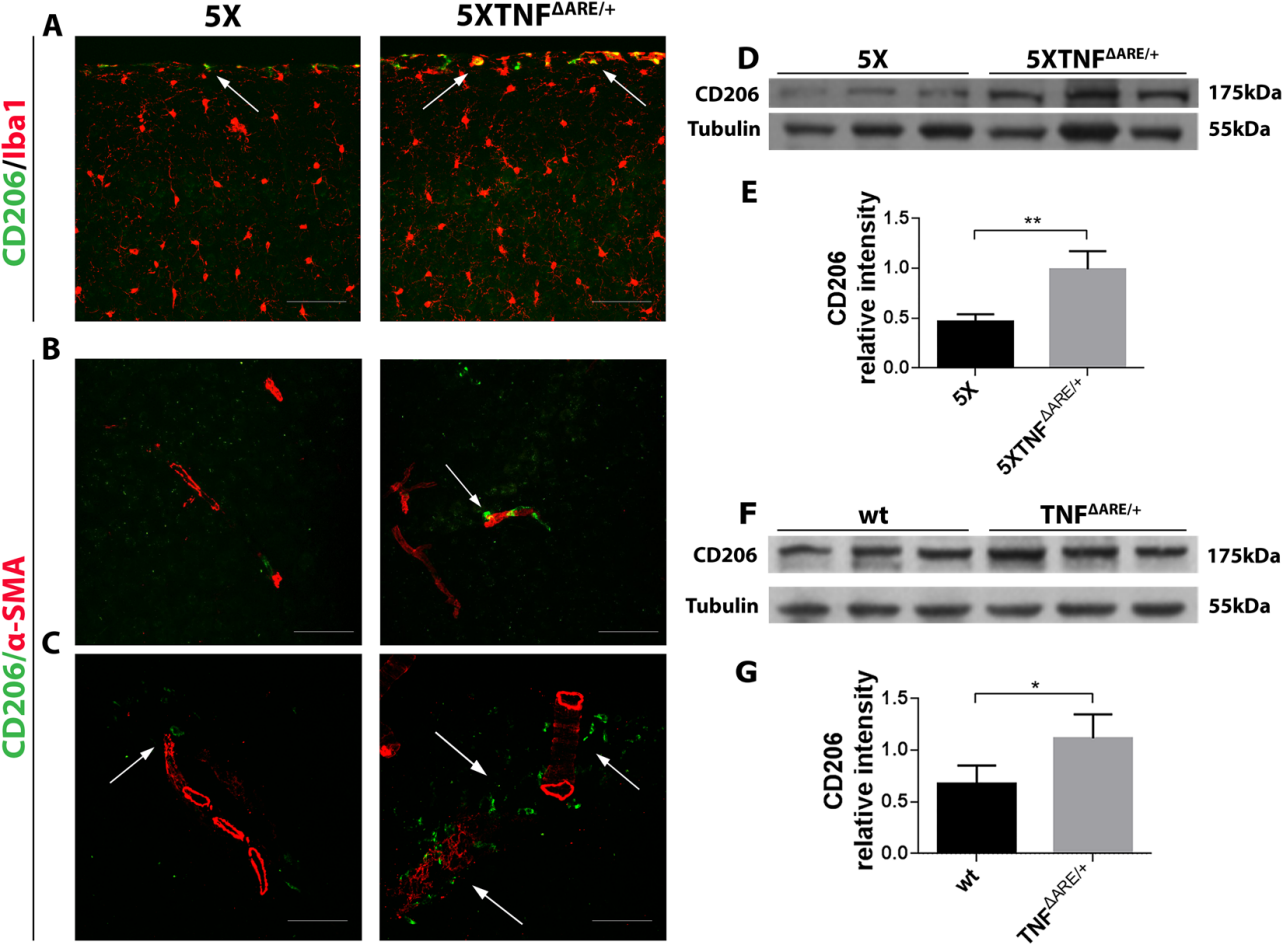
5XFAD/TNF^ΔARE/+^ mice display increased CD206-positive meningeal and perivascular macrophages (arrows). (**A – C**) Double immunofluorescence for CD206/Iba1 **(A)** and CD206/α-SMA (**B, C**) in sagittal brain sections of 4-month-old 5XFAD and 5XFAD/TNF^ΔARE/+^ mice. Representative pictures of the hippocampus, cortex and cortical vessels are shown for each group. Arrowheads indicate meningeal macrophages (**A**) and perivascular CD206-positive macrophages in the cortex (**B**) and the hippocampus (**C**). Microglia is stained red (**A**) as well as blood vessels (**B, C**) Scale bars: 25 μm. (**D-G**) Western blot analysis of CD206 brain protein levels in 4-month-old 5XFAD/TNF^ΔARE/+^ – 5XFAD (**D**) and TNF^ΔARE/+^ – C57BL/6 mice (**F**) revealed an increase in 5XFAD/TNF^ΔARE/+^ (**E**) and TNF^ΔARE/+^ mice (**G**) compared with the 5XFAD and C57BL/6 control groups. Quantitation was performed with densitometric analysis using ImageJ software. Data are mean ± SEM; *n* = 3 mice per group; experiment repeated 3 times. For statistical analyses, two-tailed unpaired *t* test was used. **p = 0.0099, *p = 0,0403.

Activation of perivascular macrophages has been associated with Aβ clearance in AD mouse models^40^. To examine whether meningeal/perivascular macrophages are activated in the 5ΧFAD/TNF^ΔARE/+^ brains, we immunostained brain sections of 5ΧFAD/TNF^ΔARE/+^ and 5XFAD control mice using a CD206-specific antibody. CD206 is a mannose receptor used as a brain perivascular macrophage marker^31,41^. Double-immunofluorescence was performed for CD206 and the microglial marker Iba1 or the blood vessel marker α-SMA. Confocal analysis revealed an increase in CD206-positive macrophages in the leptomeninges (Fig. 6A) and the perivascular space (Fig. 6B,C) of 5ΧFAD/TNF^ΔARE/+^ brains compared to the 5XFAD controls. To further examine if this a TNF-α dependent effect we also examined TNF^ΔARE/+^ and wild-type brains for CD206 protein (Fig. 6F,G). We performed Western blot analysis of CD206 levels in total brain protein extracts from 5ΧFAD/TNF^ΔARE/+^ and 5XFAD control mice (Fig. 6D) as well as TNF^ΔARE/+^ and wild-type mice (Fig. 6F). Quantitation of CD206 levels revealed a significant increase in 5ΧFAD/TNF^ΔARE/+^ mice compared the 5XFAD (Fig. 6E). Similar to 5ΧFAD/TNF^ΔARE/+^ mice, quantitation of CD206 levels in TNF^ΔARE/+^ mice displayed elevated levels compared to control mice (Fig. 6G). These findings show that the modification of the muTNF-α gene in the 5ΧFAD/TNF^ΔARE/+^ and TNF^ΔARE/+^ mice and the increase of peripheral muTNF-α can induce the activation of perivascular and meningeal macrophages.

### 5ΧFAD/TNF^ΔARE/+^ mice display significant synaptic degeneration

**Figure 7:**
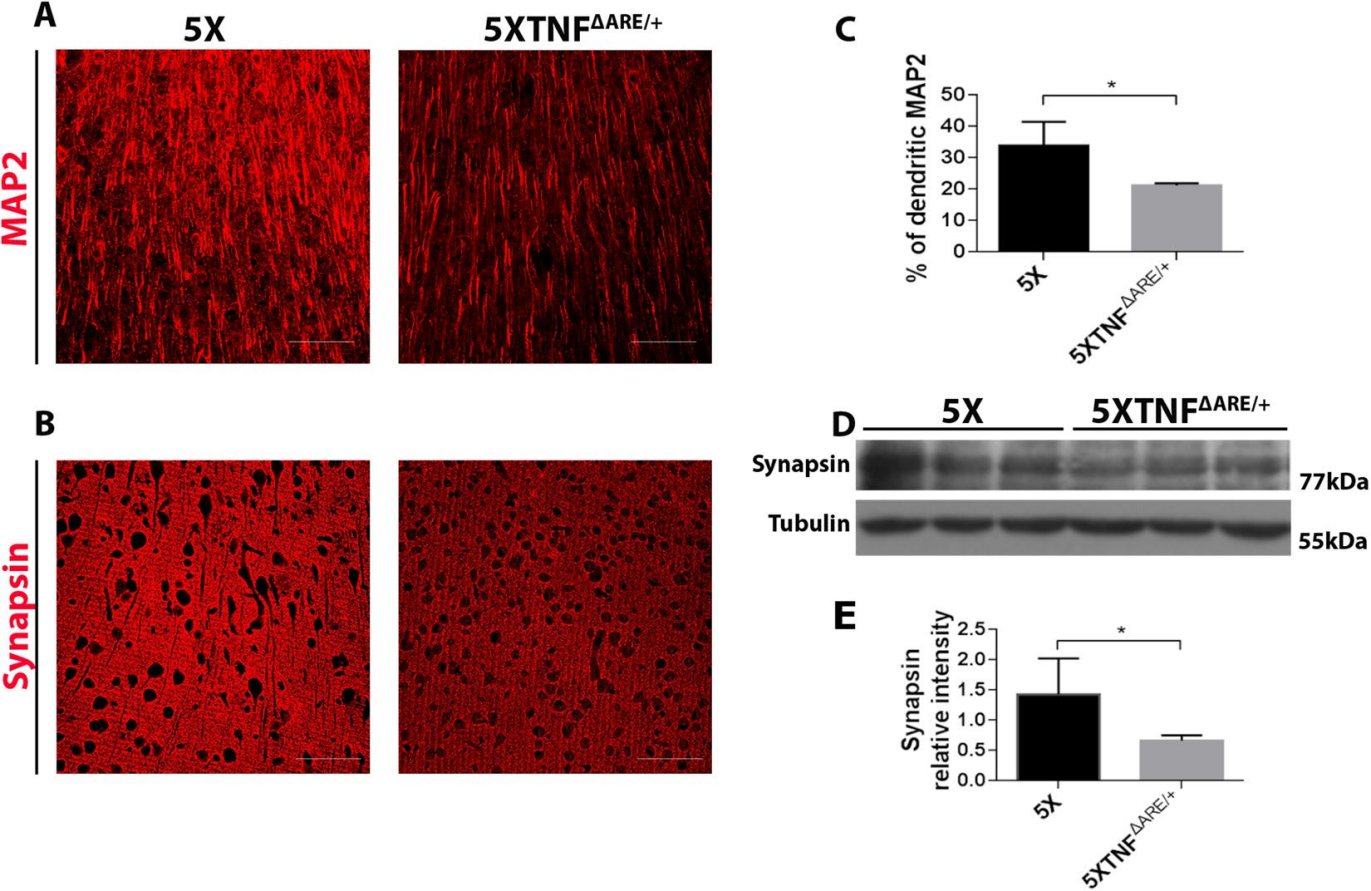
5XFAD/TNF^ΔARE/+^ mice show extensive loss of MAP2 and synapsin immunoreactivity. (**A,B**) Immunofluorescence for MAP2 (**A**) and synapsin (**B**) in sagittal brain sections of 4-month-old 5XFAD and 5XFAD/TNF^ΔARE/+^ mice. Representative pictures of the cortex are shown for each group. Scale bars, 25μm. (**C**) Quantitation of dendritic MAP2 staining in the cortex of 4-month-old 5XFAD and 5XFAD/TNF^ΔARE/+^ mice revealed a decrease in 5XFAD/TNF^ΔARE/+^ mice compared with the 5XFAD. (**D**) Western blot analysis of synapsin brain protein levels in 4-month-old 5XFAD and 5XFAD/TNF^ΔARE/+^ revealed a significant reduction of synapsin immunoreactivity in 5XFAD/TNF^ΔARE/+^ mice compared with the 5XFAD (**E**). Analysis was performed with densitometric analysis using ImageJ software. Data are mean ± SEM; *n* = 3 mice per group; experiment repeated 2 times. For statistical analyses, two-tailed unpaired *t* test was used. *p = 0.0447 (C), *p = 0,0473 (**E**).

A number of studies have suggested a role for TNF-α in synaptic function and neuronal integrity^42^. To examine the effect of the modification of the muTNF-α gene and the increase of peripheral muTNF-α in 5ΧFAD/TNF^ΔARE/+^ mice, we performed immunofluorescence for MAP2 (microtubule-associated protein 2) and synapsin in brain sections from 5ΧFAD/TNF^ΔARE/+^ and 5XFAD control mice. Confocal analysis confirmed extensive loss of MAP2 and synapsin in 5ΧFAD/TNF^ΔARE/+^ mice compared 5XFAD control mice (Fig. 7A-B). Quantitation of dendritic MAP2 immunoreactivity in the cortex of the studied mouse groups using ImageJ software confirmed a significant decrease in 5ΧFAD/TNF^ΔARE/+^ mice compared to 5XFAD (Fig. 7C). To further quantify this effect on synapsin, we performed Western blot analysis on total brain protein extracts from 5ΧFAD/TNF^ΔARE/+^ and 5XFAD mice (Fig. 7D). Similarly, to the MAP2 immunofluorescence analysis, synapsin protein levels were significantly reduced in 5ΧFAD/TNF^ΔARE/+^ mice compared to 5XFAD control mice (Fig. 7E). The observed synaptic loss in 5ΧFAD/TNF^ΔARE/+^ mice suggest for a detrimental effect on neuronal health resulting from the modification of the muTNF-α gene and the increase of peripheral muTNF-α levels.

## Discussion

In the present study, we investigated the role of TNF-α in the development of the amyloid phenotype in an AD mouse model by modulating the 3′UTR of the endogenous mouse gene. We employed a widely used AD mouse model, the 5XFAD^26^, and a TNF-α knock-in transgenic mouse with deletion of the AU-rich elements in the 3′UTR of the endogenous mouse TNF-α (TNF^ΔARE/+^) that results in elevated mouse TNF-α levels in the blood and development of RA and Crohn’s disease^25^. Analysis of the 5XFAD/TNF^ΔARE/+^ mice showed that the genetic modification of the TNF-α endogenous gene protects against the amyloid phenotype in the mouse brain. 5XFAD/TNF^ΔARE/+^ brains exhibit significantly decreased amyloid deposition and increased microglial activation. Perivascular macrophages and infiltrating leukocytes are also increased. Astrocytic activation in the brain is not affected and APP processing enzymes remain in the same levels compared to 5XFAD mice. Interestingly, peripheral TNF-α levels are increased in the 5XFAD/TNF^ΔARE/+^ mice compared to 5XFAD, while brain TNF-α levels are not affected.

TNF-α is a major pro-inflammatory cytokine that has been identified as a key molecule involved in autoimmune diseases as RA and neurodegenerative diseases as AD^6^. RA is a chronic autoimmune disease driven by TNF-α^13^. RA patients receiving anti-TNF-α drugs show reduced risk of developing AD^20^, raising questions about the role of RA and/or anti-TNF-α therapy in AD. Despite numerous studies focusing on the therapeutic role of TNF-α in AD and the potential protective effects of anti-TNF drugs, the underlying mechanisms are still unknown^43–46^. In this study, analysis of 4-month-old 5XFAD/TNF^ΔARE/+^ mice that develop an AD-like phenotype, revealed a significant decrease of Thioflavine-S positive plaques and Αβ deposition in mouse brains compared to the 5XFAD controls, suggesting that the modification of the 3′UTR of the muTNF-α endogenous gene, exerts a protective effect against amyloid deposition in the brain. This result is similar to our previous study^21,22^, where 5XFAD mice carrying a human TNF-α transgene with a modified 3′UTR^23^ developed reduced amyloid deposits and robust brain inflammation. In both studies, alteration of the 3′UTR of the TNF-α gene, resulted in decreased amyloid plaque formation in the brain. Interestingly the same result, is observed by blocking the TNF-α signaling pathway either by genetically eliminating TNF-α or the p55 TNF-α receptor in AD mice^47,48^. Genetic deletion of TNF-α is protective against amyloid plaque formation and Αβ production in AD mice, by decreasing amyloidogenic APP enzymatic processing and Aβ production^47^. Deletion of the p55 receptor in AD mice also reduces amyloid plaque deposition by decreasing Aβ production^48^, whereas genetic deletion of the p75 receptor exacerbates amyloid deposition^49^ suggesting a complex role for TNF-α signaling pathway in AD pathogenesis. To elucidate the mechanism involved in the reduction of amyloid deposits in 5XFAD/TNF^ΔARE/+^ mice, we first looked for differences in APP protein levels and the APP processing enzymes involved in Aβ production between 5XFAD/TNF^ΔARE/+^ and control 5XFAD brains. Our analysis did not show any differences in APP protein levels or the APP processing enzymes between the two groups. BACE1, which is the β-secretase involved in Aβ generation^50^ as well as TACE, and ADAM10, two of the α-secretases that cleave APP^51–54^, did not show any significant changes in their protein levels in the mouse brains. Previous studies, including ours^47,48^, have shown that genetic deletion of TNF-α or the TNF-α receptors’, p55 and p75, can modulate α- and β-secretase protein levels in AD mice, affecting Aβ production and amyloid plaque deposition^50,51^. Since TNF-α has been implicated in APP cleavage by modulating PS1, a component of the γ-secretase complex, through the JNK-dependent pathway^29,30^ we also looked for differences in protein levels of PS1, as well as Nicastrin and Aph1, proteins of the γ-secretase complex, in the brains of 5XFAD/TNF^ΔARE/+^ mice. Our analysis for PS1, Nicastrin and Aph1 showed no difference in protein levels between 5XFAD/TNF^ΔARE/+^ and control brains. Overall, our analysis of the APP processing enzymes in the 5XFAD/TNF^ΔARE/+^ mice provide evidence that modification of the 3’UTR of the TNF-α gene does not affect APP processing or Aβ production in the brain.

Microglia and astrocytes are the immune cell populations of the brain involved in Aβ clearance. Since 5XFAD mice display increased microglial and astrocytic activation associated with amyloid deposition^26^, we examined the glial response in the 5XFAD/ TNF^ΔARE/+^ brains. Our analysis revealed that modification of the 3′UTR of the TNF-α mouse gene results in a robust increase in microglial activation with a small increase in phagocytic and autophagy markers as CD68 and LC3 in the 5XFAD/TNF^ΔARE/+^ brains. To further analyze this result, we examined brains of TNF^ΔARE/+^ mice where we also found a robust increase of microglial activation, which suggests that modification of the 3′UTR of the muTNF-α gene is responsible for the microglial activation in the 5XFAD/TNF^ΔARE/+^ brains. This finding is similar to our previous study^21^ where transgenic mice expressing a human TNF-α transgene with a modified 3′UTR also showed highly activated microglia with increased phagocytic capacity. Our data, together with other studies where genetic elimination of the TNF-α gene or the p55 receptor gene, or pharmacological inhibition of TNF-α suppresses microglial activation^47,48^, suggest a key role for TNF-α in modulating microglial response. Next, we analyzed astrocytic activation in the 5XFAD/TNF^ΔARE/+^ and 5XFAD control brains and, we found no differences between the two groups. Further analysis between TNF^ΔARE/+^ and control brains confirmed this finding suggesting that modification of the 3′UTR of the muTNF-α gene does not affect the activation status of the astrocytes in the physiological or the AD brain context. Similarly, to this finding, previous studies in mice using LPS induction and inhibition of the TNF-α signaling with Etanercept in the CNS, have confirm specific activation of microglia and not astrocytes^55^. These findings suggest that increased levels of muTNF-α either from acute (LPS)^55^ or chronic induction (expression of muTNF-α in TNF^ΔARE^ mice) might not be sufficient or capable to activate astrocytes. Interestingly, this is in contrast to our previous study where modification of the 3′UTR of a human TNF-α transgene resulted in robust astrocytic response in the mouse brain^21^. The increased astrocytic activation observed in our previous study, might be related to the activation level of microglia^56^ as in the huTNF-α expressing brains^21^ microglia is highly activated throughout the brain, in comparison to the muTNF-α expressing TNF^ΔARE^ brains, where microglial activation was milder. These data imply for a possible differential activation of the two glial cell types, microglia and astrocytes, by TNF-α and further studies are needed to investigate this effect. Overall, our results suggest for a central role of microglia in restricting the amyloid deposits in the mouse brains.

Modification of the 3′UTR of the TNF-α gene results in significant increase in the levels of peripheral TNF-α in the TNF^ΔARE/+^ mutant mice^25^. To evaluate this effect in 5XFAD/TNF^ΔARE/+^ mice we measured TNF-α levels in the serum of mice as well as in brain protein extracts. Our analysis revealed a significant increase in TNF-α levels only in the periphery of 5XFAD/TNF^ΔARE/+^ mice compared to 5XFAD controls, in similar levels to our previous study^21^ (~200 pg/ml) while brain TNF-α remained the same. This result suggests that, similarly to our previous study^21^, modification of the 3′UTR of the TNF-α gene, can result in significantly increased TNF-α levels in the periphery, while brain levels remains unchanged. To further evaluate whether this increase in peripheral TNF-α can affect bone marrow derived cell populations involved in Αβ clearance^41^, as brain infiltrating leukocytes and perivascular and meningeal macrophages, we looked for CD45 and CD206 positive cells in the 5XFAD/TNF^ΔARE/+^ brains. Our analysis revealed a significant increase of CD45 positive leukocytes in the brain as well as CD206-positive meningeal/perivascular macrophages in the 5XFAD/TNF^ΔARE/+^ mouse brains compared to the 5XFAD. A previous study by D′ Mello *et al*., has supported the central role of TNF-α during peripheral inflammation in orchestrating the periphery to brain communication pathway^32^. CD45 positive leukocytes that infiltrate the brain, have been identified as a key cell population involved in this pathway together with activated microglia. The abovementioned study together with our previous^21^ and current study support CD45 leukocytes as a mediator and potent regulator, between the peripheral immune system and the brain’s innate immune cell type, microglia. Furthermore, CD206 perivascular macrophages, that are known to participate in Aβ clearance^40,41^ are also activated in the mouse brains. Our analysis support CD45 and CD206 positive cells as the mediators of the effects of peripheral TNF-a into the 5XFAD/TNF^ΔARE/+^ mouse brains.

Our analysis for MAP2 and synapsin, cell markers that reveal neuronal and synaptic health, shows that 5XFAD/TNF^ΔARE/+^ mice display a clear reduction for both. Several lines of evidence have suggested that TNF-α regulates neuronal and synaptic health in the brain^42^. Neuronal TNF-α expression in AD mice results in neuronal death and extensive microglial activation^57^ and TNF-induced neuronal and synaptic loss is mediated by microglial phagocytosis^58,59^. These findings suggest for microglial activation resulting from peripheral TNF-α as a significant modulator of neuronal and synaptic integrity.

We and others have shown that interfering with the TNF-α signaling pathway can modify the AD-like phenotype in the brain of AD mouse models^21,47–49,60^. Transgenic mice that carry modified TNF-α or TNF-α receptors genes have helped to better comprehend the role of TNF-α in the AD pathogenetic mechanisms. These studies have been sometimes conflicting and not easy to interpret, suggesting a complex effect of TNF-α on the parameters involved in AD pathogenesis. We and others have found that inactivating the TNF-α signaling pathway by genetically deleting the TNF-α gene or the TNF-α receptor p55 gene results in decreased amyloid deposition in the mouse brain^47,48^. On the contrary genetic deletion of the TNF-α receptor p75 gene or both receptors p55 and p75 results in increased amyloid deposition suggesting a complex mechanism involving the TNF-α signaling pathway^49,60^. In our previous study overexpression of a huTNF-α modified transgene in AD/arthritic mice (5XFAD/Tg197) has resulted in a brain phenotype with exacerbated neuroinflammation and reduced amyloid deposition^21^. Surprisingly this brain phenotype could be modified by regulating peripheral huTNF-α levels with the use of an anti-TNF-α therapeutic antibody for RA, administered at the periphery, thus confirming the key role of peripheral TNF-α. To further investigate the role of muTNF-α in the AD pathogenetic mechanism in the 5XFAD mice, in the current study, we employed a TNF-α knock-in mouse, the TNF^ΔARE/+^, where the effects observed on the AD-like phenotype are mediated by muTNF-α. These effects mediated by the mouse TNF-α differ from the previously characterized effects of a human TNF-α transgene^21^. Specifically, the brain inflammatory phenotype in 5XFAD/TNF^ΔARE/+^ mice is milder with less activated microglial cells and lacks astrocytic activation compared to the 5XFAD/Tg197. Yet, in both studies a significant reduction in amyloid plaque formation is observed that coincides with activated microglia and synaptic degeneration, suggesting a central role for microglia in modulating the AD-like phenotype. A possible explanation for this difference in the glial activation between the two studies could be that in the 5XFAD/Tg197 brains, where mice express the human TNF-a, the mouse p75 receptor is unable to bind the human TNF-α^61^. Therefore, in 5XFAD/Tg197 brains, huTNF-α binds only p55 and mediates its effects through this receptor only, while in 5XFAD/TNF^ΔARE/+^ brains, muTNF-α binds both p55 and p75 receptors. Previous studies involving traumatic brain injury, have suggested for a protective role for p75 in brain health, while the role p55 has been detrimental^62^. Further supporting the protective role of p75 in the brain, Etanercept, a fusion protein of p75, used in RA treatment, has shown promising results in AD treatment^20^. Finally in agreement with our previous study^21^, in 5XFAD/TNF^ΔARE/+^ brains, CD45 and CD206 positive cell activation remains strong supporting the role of these bone-marrow derived cell populations in mediating the effect of peripheral TNF-α.

Our current and previous studies dissociate the human and the mouse TNF, in modulating neuroinflammation, in an AD/TNF transgenic mouse system. Our findings suggest for a potent protective role of the p75 TNF receptor in modulating brain inflammation and point out the TNF pathway as a major therapeutic target in AD therapeutics. More elaborate studies that involve genetic or pharmaceutical manipulation of the p75 TNF receptor in AD/TNF transgenic mice will help to further elucidate the underlying mechanism.

## Methods

### Animals

5XFAD mice^26^ were purchased from The Jackson Laboratory (Bar Harbor, ME, USA) and were on a C57Bl6/J genetic background. TNF^ΔARE/+^ mice^27^ were kindly provided by Dr. G.Kollias (Fleming Institute, Greece) and were on a C57BL/6 J genetic background. Both 5XFAD and TNF^ΔARE/+^ transgenic lines were maintained as hemizygotes. For the purposes of this study, 5XFAD hemizygous mice were crossed with TNF^ΔARE/+^ mice to generate 5XFAD/TNF^ΔARE/+^ double transgenic mice along with 5XFAD, TNF^ΔARE/+^ and wild-type non-transgenic littermates used as controls. Only female mice were used in the analysis, as 5XFAD females develop the amyloid phenotype 2 months earlier than 5XFAD males^26,28^. Mouse genotyping was performed with polymerase chain reaction. All mice were housed in the SPF facility of the Biomedical Research Foundation and maintained on a standard chow diet containing 5% fat (Teklad; Harlan). All animal procedures were approved by the Bioethical Committee of the Biomedical Research Foundation and were in agreement with ethical recommendations of the European Communities Council Directive (86/609/EEC). The approved procedures for animal care and treatment were in accordance with the Association for the Assessment and Accreditation of Laboratory Animal Care (AAALAC) and the recommendations of Federation of European Laboratory Animal Science Association (FELASA).

### Tissue collection

Mice were anesthetized and transcardially perfused with ice-cold PBS as previously described^21,28,40,47,63^ After euthanasia, the brains were removed and cut along the sagittal midline. Left hemibrains were snap-frozen for protein analysis. Right hemibrains were immersion-fixed in PBS-buffered 4% paraformaldehyde (Sigma Aldrich) for 48 hours and then cryoprotected in 20% sucrose in PBS for histological analysis.

### Tissue processing for protein extraction

Protein extraction from hemibrains was carried out in sequential steps as previously described^21,28,40,47,63^. Tissues were homogenized in ice-cold PBS, containing protease inhibitors (Complete Mini Protease Inhibitor Cocktail Tablets, Roche Diagnostics) with a Tissue homogenizer (Wheaton). The homogenate was centrifuged at 12500 rpm (Biofuge fresco microcentrifuge, Fixed-angle rotor 7500 3325, Heraeus) for 45 min at 4 °C. The supernatant (PBS fraction) was removed and used to evaluate Iba1 and CD206 protein levels. The pellets were resuspended in ice-cold lysis buffer (containing 10% glycerol, 1% Triton X-100 and protease inhibitors in PBS) and centrifuged at 9000 rpm for 10 min at 4 °C. The supernatant (lysis fraction) was removed and used to evaluate APP/CTFs, BACE1, ADAM10, TACE, PS1/CTF, Nicastrin, Aph1, synapsin and GFAP protein levels.

### Western blot analysis

Analysis was performed as previously described^21,28,40,47,63^. Specifically, protein concentrations of all samples were determined using the BCA Protein Assay Kit (Thermo Fisher Scientific, Waltham, MA, USA) according to the manufacturer’s protocol. Equal amounts of total protein from hemibrains were separated on SDS-PAGE 8–16% Tris-Glycine gels (or 12% Tris-Tricine gels for APP/CTFs and Iba1) and electrophoretically transferred to nitrocellulose membrane (Protran, Whatman). For the detection of PS1/CTF and APP/CTFs, proteins were transferred to PVDF membrane (Merck Millipore, Darmstadt, Germany). Membranes were cut in two pieces, one piece, containing protein extracts of molecular weight approximately around 50 kDa, was processed separately for tubulin, and the other half was incubated separately with each of the below mentioned primary antibodies. Only for the detection of GFAP that has a molecular weight close to tubulin (50 kDa GFAP-55kDa tubulin), membranes were first processed for detection of GFAP and then stripped (with 1,5% w/v Glycine, 0,1%w/v SDS, 1% v/v Tween-20 pH=2,2) and re-incubated for tubulin. Specifically, membranes were blocked with 3–5% non-fat milk in TBS/Tween-20 0.05–0,1% and then incubated with specific primary antibodies: mouse monoclonal anti-BACE1 (1:300; Merck Millipore), anti-PS1 C-terminal (1:500; Merck Millipore), anti-GFAP (1:1500; Sigma Aldrich) and anti-tubulin (1:500; Sigma Aldrich), rat anti-CD206 (1:250, AbD Serotec), goat polyclonal anti-TACE (1:300; Santa Cruz Biotechnology) and anti-Nicastrin (1:1000; Santa Cruz Biotechnology) and rabbit polyclonal anti-APP C-terminal (1:1500; Sigma Aldrich), anti-ADAM10 (1:500; Chemicon), anti-Aph-1 (1:300; Merck Millipore), anti-synapsin (1:1000; Cell Signaling), anti-Iba1 (1:500; Wako, Osaka, Japan) and anti-LC3 (1:1000; Merck) Membranes were then incubated with the corresponding HRP-conjugated secondary antibody (1:3000–1:5000; Santa Cruz Biotechnology) and developed using enhanced chemiluminescence. Densitometric analysis was performed using the NIH Image J software.

### Enzyme-linked immunosorbent assays (ELISA)

For muTNF-α quantitation by ELISA, serum and PBS brain homogenates were diluted with the ELISA sample buffer and sample duplicates were run on specific sandwich colorimetric ELISAs (Biolegend) according to the manufacturer’s protocol as previously described^64^. Optical densities at 450 nm of each well were read on a microplate reader (ELx800, BioTek Instruments, Winooski, VT, USA), and sample TNF-α concentrations were determined by comparison with the respective standard curves using the Gen5 software (BioTek Instruments). Brain sample values were normalized to total brain protein concentrations determined using the BCA Protein Assay Kit. The results were calculated as mean ± SEM for each genotype.

### Thioflavine-s staining

Analysis was performed as previously described^21,28,40,47,63^. Specifically, fixed hemibrains were cut in 40μm sagittal free-floating sections from the genu of the corpus callosum to the most caudal hippocampus using a vibratome (Leica VT1000S, Leica Microsystems). For the quantitation of amyloid plaque load, sections were incubated for 9 minutes in 1% w/v Thioflavine-S (Sigma Aldrich) aqueous solution and then differentiated 2 times with 80% ethanol for 3 min each, followed by another 3 min wash with 95% ethanol. Sections were rinsed three times with ddH_2_O and coverslipped with mounting medium for fluorescence (Vectashield, Vector Laboratories). Imaging for Thioflavine-S was performed on a Leica TCS SP5 confocal microscope and image captivation was performed using the Leica LAS AF Suite. Images for Thioflavine-S load quantitation were captured using HCImage software (Hamamatsu) in a Leica DMRA 2 microscope.

### Αβ immunohistochemistry

Analysis was performed as previously described^21,28,40,47,63^. Specifically, 40μm sagittal free-floating sections were permealized with TBS/Triton X-100 0.1% 3 times for 10 min each, incubated for 30 min with 0.6% H_2_O_2_ in TBS and washed 3 times 5 min each with TBS. Antigen retrieval was performed with 98% formic acid (AppliChem) for 5 min, followed by 3 washes with TBS. Sections were blocked in 15% normal goat serum (Vector Laboratories) in TBS/Triton X-100 0.1% for 1 hour and incubated overnight at 4^ο^C with the mouse monoclonal biotinylated 6E10 antibody (1:500, Covance, Princeton, NJ, USA) in 5% normal goat serum (Vector Laboratories) in TBS/Triton X-100 0.1%. Sections were then washed 4 times with TBS plus one final wash with PBS, and incubation in avidin-biotinylated horseradish peroxidase complex (Vectastain Standard ABC kit, Vector Laboratories) followed for 120 min at room temperature. Peroxidase labeling was visualized with DAB/Ni (peroxidase substrate kit, Vector Laboratories). After a 1–2 min incubation period, sections were washed with ddH_2_O, mounted on polylysine-coated slides (Thermo Fisher Scientific), dehydrated in increasing ethanol concentrations from 50 to 100% followed by xylene (Sigma Aldrich) and coverslipped with DPX mounting medium (AppliChem). Imaging was performed with a DM LS2 Leica microscope, and image capture was carried out using the Leica Application Suite (version V4.6).

### Immunofluorescence

Analysis was performed as previously described^21,28,40,47,63^. For immunofluorescent labeling, 40μm sagittal free-floating sections were first subjected to three 5 min washes with PBS and antigen retrieval with 10 mM sodium citrate buffer pH 6, for 30 min at 80 °C. The sections were washed again with PBS, then blocked for 1 h in 10% FBS (Thermo Fisher Scientific), 1% BSA (Sigma Aldrich) in PBS/Triton X-100 0.3% and incubated overnight at 4 °C with rat anti-CD206 (1:250; AbD Serotec), rabbit anti-Iba1 (1:500; Wako), rabbit anti-smooth muscle actin (α-SMA) (1:250; GeneTech), rabbit anti-synapsin (1:1000; Cell Signaling), rat anti-CD45 (1:200; ImmunoTools) and rat anti-CD68 (1:250; AbD Serotec) antibodies in 1% FBS, 1% BSA in PBS/Triton X-100 0.3%. Sections were washed with PBS and developed with anti-rabbit Cy3-conjugated (1:500; Jackson ImmunoResearch, West Grove, PA, USA), anti-rat Alexa-633-conjugated (1:500; Invitrogen) and anti-mouse Alexa-546-conjugated (1:500; Invitrogen) secondary antibodies for 1 h at room temperature. Where needed, sections were treated with 1% Thioflavine-S aqueous solution for 5 min, differentiated twice in 70% ethanol and washed in PBS. For GFAP and Thioflavine-S double labeling, sections were blocked for 1 h in TBS/Triton X-100 0.4% supplemented with 5% normal goat serum and immunolabeled with anti-GFAP (1:500; Sigma Aldrich) antibody in the blocking solution overnight at 4 °C. Sections were washed with TBS, developed with an anti-mouse Alexa-546-conjugated secondary antibody (1:500; Invitrogen) for 1 h at room temperature and then processed for Thioflavine-S staining. For MAP-2 immunofluorescent detection, sections were permeabilized for 1 h in PBS/Triton X-100 0.3%, blocked for 1 h in 10% FBS, 1% BSA in PBS/Triton X-100 0.3%, and incubated overnight at 4 °C with a rabbit anti-MAP-2 (1:100; Santa Cruz Biotechnology) antibody in 1% FBS, 1% BSA in PBS/Triton X-100 0.3%. Sections were washed with PBS and processed with an anti-rabbit Cy3-conjugated (1:500; The Jackson Laboratory) secondary antibody for 1 h at room temperature. All sections were finally mounted on polylysine-coated slides and covered with mounting medium (Vectashield). Imaging was performed on a Leica TCS SP5 confocal microscope and images were captured using the Leica LAS AF Suite.

### Image analysis, quantitation and statistics

All confocal microscopy images were taken using the Leica Application Suite, Advanced Fluorescence Lite, 263 build 8173 software (Leica Microsystems). All images were analyzed using the Image J software (NIH) as previously described^21,28,40,47,63^. For Thioflavine-S load quantitation, six 40μm sagittal hippocampal sections 240μm were chosen. Images were analyzed with the NIH Image J software in an 8-bit grayscale format. Figures were generated using adobe. photoshop. elements 12 Version 12.1 (20140303.12.1.49334) software. Hippocampal or cortical area was outlined manually, and the surrounding area was cleared. Thioflavine-S load was expressed as the percent area covered by Thioflavine-S positive plaques (% Thioflavine-S)^21,28,40,47,63^. For the quantitation of cortical CD45^high^ leukocytes, Three 40μm sagittal sections 240μm apart from each other per brain (n = 3) were immunostained for CD45 and 20x magnification image stacks were captured on a Leica TCS SP5 confocal microscope. Cell counting was performed using the Cell Counter Image J plugin as previously described^21^. For the quantitation of microtubule-associated protein 2 (MAP2) immunoreactivity, six 40 μm sagittal sections 240 μm apart from each other per mouse brain were immunostained and 40x magnification image stacks were captured on a Leica TCS SP5 confocal microscope. The percentage area covered by MAP2-positive dendrites was measured (% of dendritic MAP2)^21^. All the obtained measurements per section were calculated as the mean value for each mouse brain and used for statistical analysis. Statistical analyses were performed using GraphPad Prism (version 6; GraphPad software Inc., La Jolla, CA, USA) as previously described^21,28,40,47,63^. All data were analyzed using two-tailed unpaired t-test and expressed as mean ± SEM. Statistical significance was defined as *P* < 0.05.

## Supplementary information

Supplementary information.
