# Supplementary material for: A 3′UTR modification of the TNF-α mouse gene increases peripheral TNF-α and modulates the Alzheimer-like phenotype in 5XFAD mice
Source: Sci Rep. 2020 May 26;10:8670. doi: 10.1038/s41598-020-65378-2 (PMC7250826; doi:10.1038/s41598-020-65378-2)

A 3'UTR modification of the TNF-α mouse gene increases peripheral TNF-α and modulates the Alzheimer-like phenotype in 5XFAD mice.

Nikoleta Kalovyrna[1], Olympia Apokotou[1], Sotiria Boulekou, Evi Paouri, Athena Boutou, and Spiros Georgopoulos*

[1] These authors contributed equally to this work.

Figure 2 G

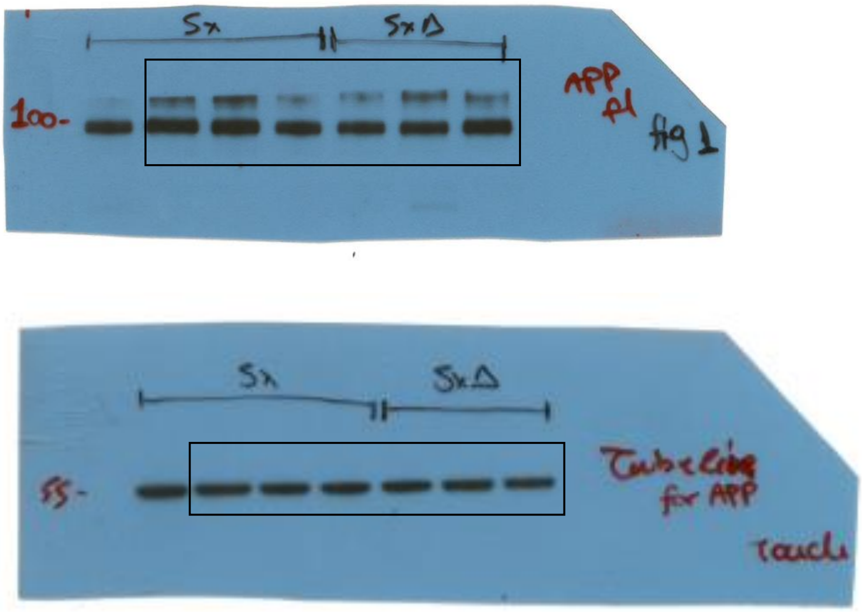

Figure 3 A

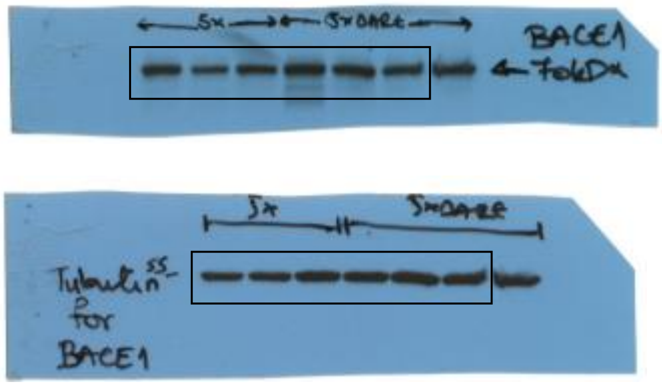

Figure 3 C

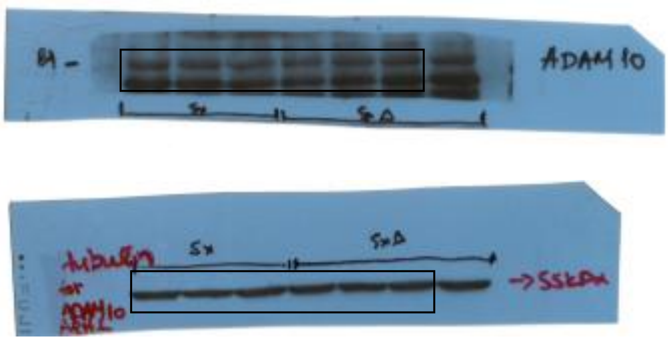

Figure 3 E

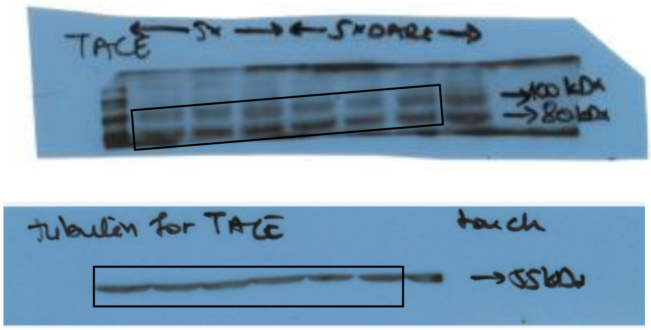

Figure 3 G

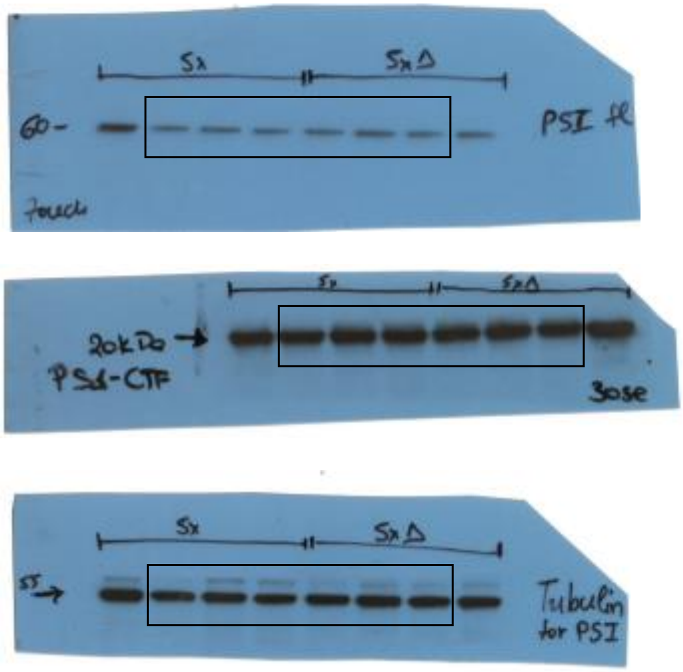

Figure 3 J

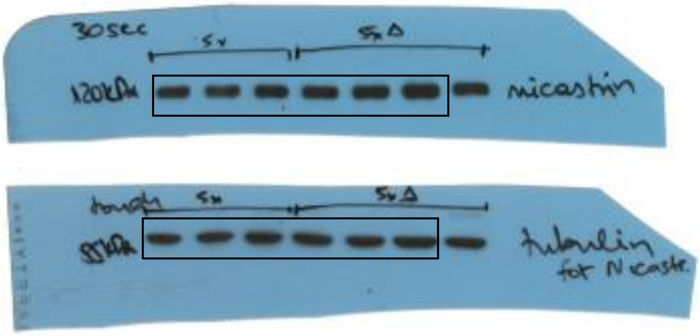

Figure 3 L

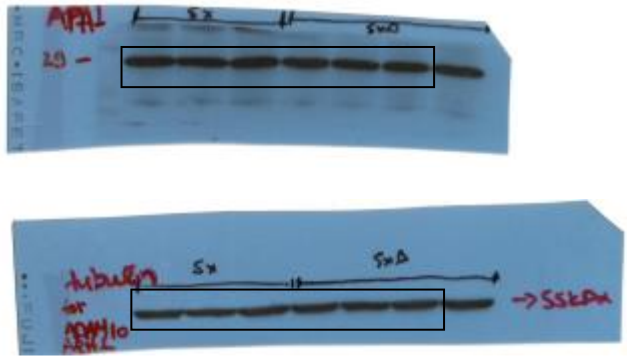

Figure 4 B

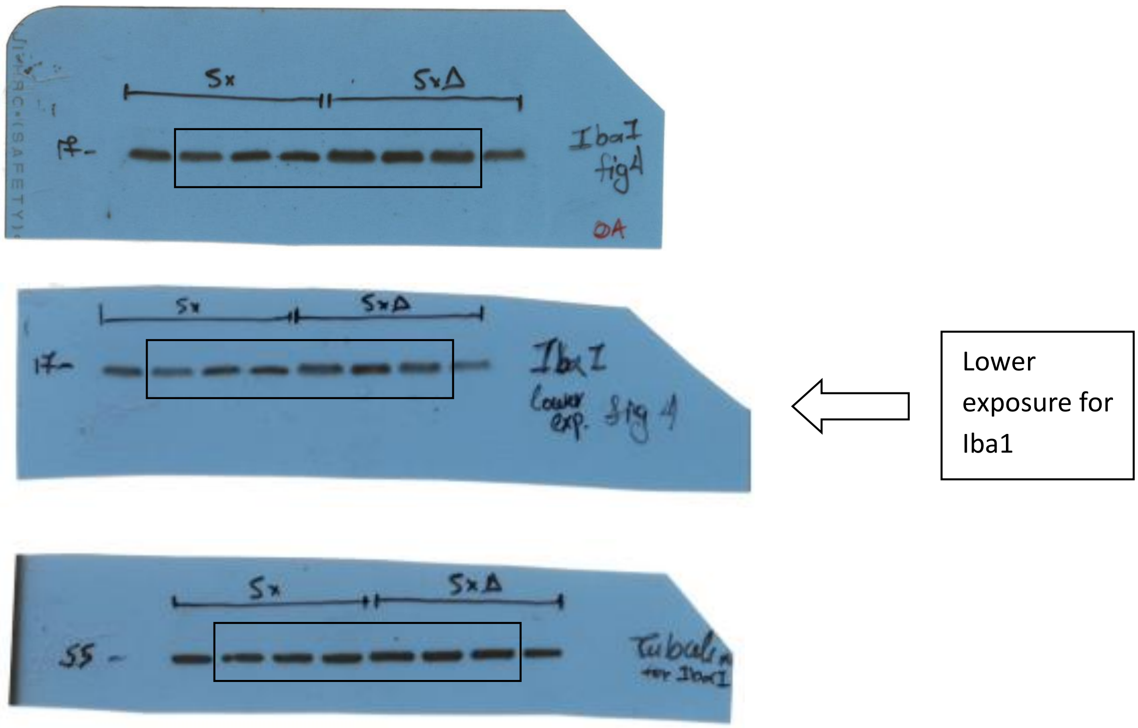

Figure 4 E

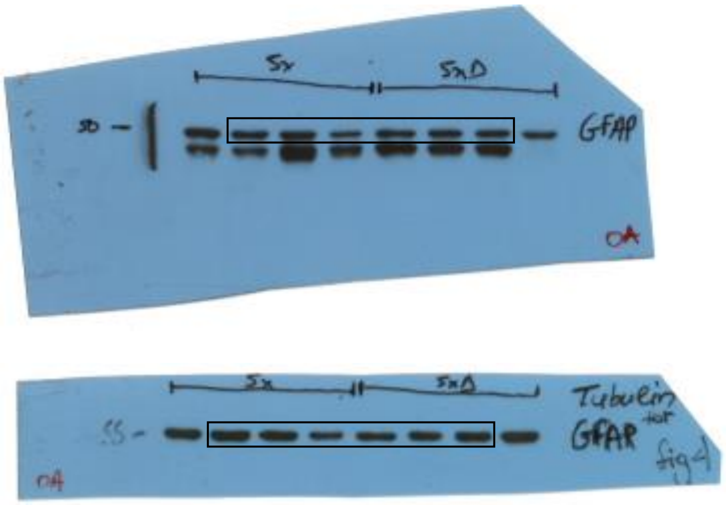

Figure 4 G

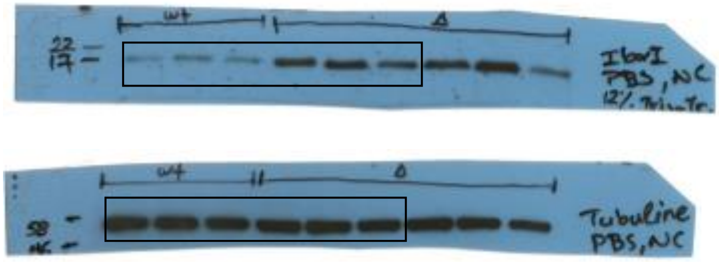

Figure 4 I

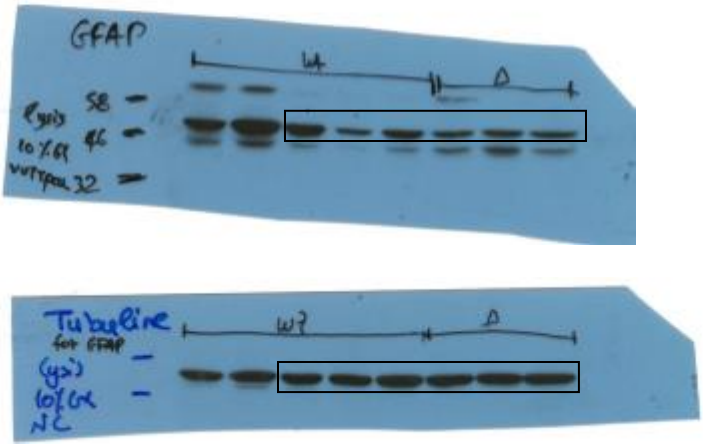

Figure 5 E

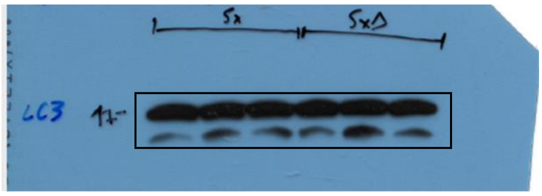

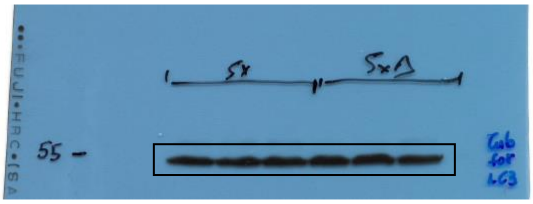

Figure 6 D

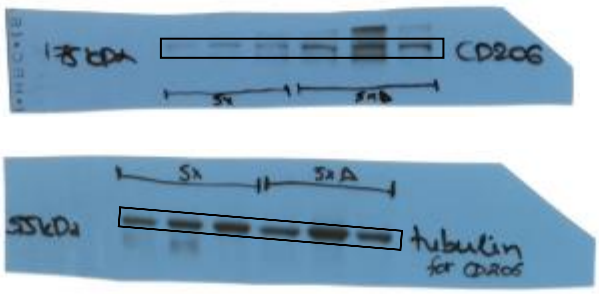

Figure 6 F

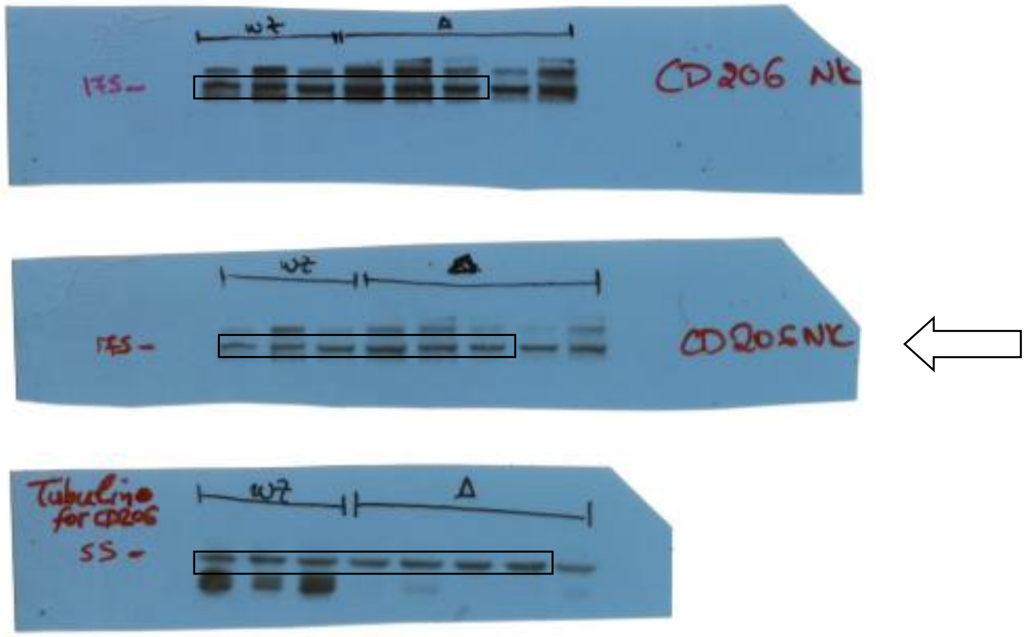

Lower exposure for CD206

Figure 7 D

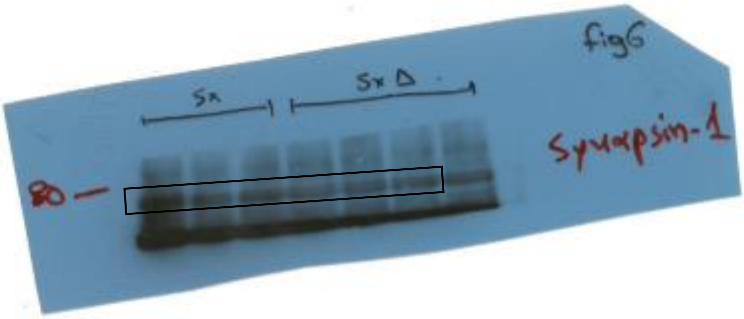

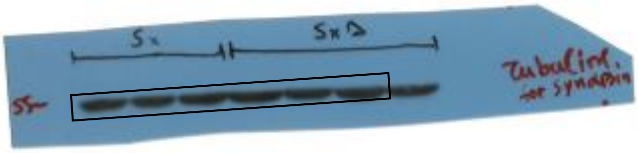

Supplement: Supplementary file 1 — Supplementary information. [file 41598_2020_65378_MOESM1_ESM.pdf]
